# Case report of two long term ovarian cancer survivors with brain metastases following multimodal treatment including chemotherapy, radiotherapy and maintenance olaparib: An institutional case series and literature review

**DOI:** 10.1016/j.gore.2024.101444

**Published:** 2024-06-24

**Authors:** Yukari Tsuchino, Tatsuyuki Chiyoda, Mitsuyo Jisaka, Tomomi Sakamaki, Momo Hirata, Mio Takahashi, Takuma Yoshimura, Kensuke Sakai, Michiko Wada, Wataru Yamagami

**Affiliations:** Department of Obstetrics and Gynecology, Keio University School of Medicine, Tokyo, Japan

**Keywords:** Ovarian cancer, Brain metastasis, PARP inhibitors, Olaparib

## Abstract

Brain metastasis from ovarian cancer is a very rare condition with a poor prognosis. However, due to its rarity, there is no established treatment strategy. We present a case series of brain metastasis with ovarian cancer, focusing on two long-term survivors treated with multimodal therapy. Among the nine cases, the median survival time after brain metastases was six months (range: 0–58 months). Eight patients had high-grade serous carcinoma (HGSC). Three of the four patients who underwent genetic testing tested positive for germline *BRCA2* (g*BRCA2*) mutation. Two patients survived longer than 4 years after the diagnosis of brain metastases. Both of these patients received chemotherapy, radiation therapy, and olaparib, a molecularly targeted drug, as maintenance therapy. This case series suggests that patients with g*BRCA2* mutation-positive HGSC may be at a high risk of developing brain metastases. A multidisciplinary approach, including PARP inhibitors, may improve the prognosis of patients with brain metastases from ovarian cancer.

## Highlights

• Brain metastases from ovarian cancer are rare and have a poor prognosis.
• A multidisciplinary approach including radiation therapy, chemotherapy, and PARP inhibitors may improve the prognosis of patients with brain metastases from ovarian cancer.
• Three of the four patients (75%) with brain metastases who underwent genetic testing had g*BRCA2* mutation.

## 1 Introduction

Brain metastasis from ovarian cancer is a rare condition, occurring in only 1.34 % of all ovarian cancer patients. It has a poor prognosis and is characterized by a survival time of approximately 10.1 months after brain metastasis (Borella et al., 2020). For decades, local therapies such as surgery and radiation have been the mainstay of treatment for brain metastases from solid tumors, primarily due to the lack of systemic therapies demonstrating meaningful intracranial activity. Furthermore, clinical trials have typically excluded patients with brain metastases, thereby limiting the available evidence regarding the intracranial efficacy of systemic therapies. However, in melanoma, lung cancer, and breast cancer, novel systemic therapies, such as immunotherapy and targeted therapies, have shown promising efficacy in controlling intracranial disease (Amouzegar et al., 2024).

However, given its rarity, there is currently no established treatment strategy for brain metastases from ovarian cancer. This case series aims to clarify treatment strategies for patients with brain metastases originating from ovarian cancer.

## 2 Method

Between January 2012 and August 2023, we conducted a retrospective analysis of clinicopathological factors, treatment modalities for brain metastasis, and post-metastasis prognosis in nine cases of ovarian cancer-related brain metastasis at our institution. Overall survival was defined as the time from the initial diagnosis to the date of death or confirmed survival.

## 3 Results

### 3.1 Case 1

**Fig. 1:**
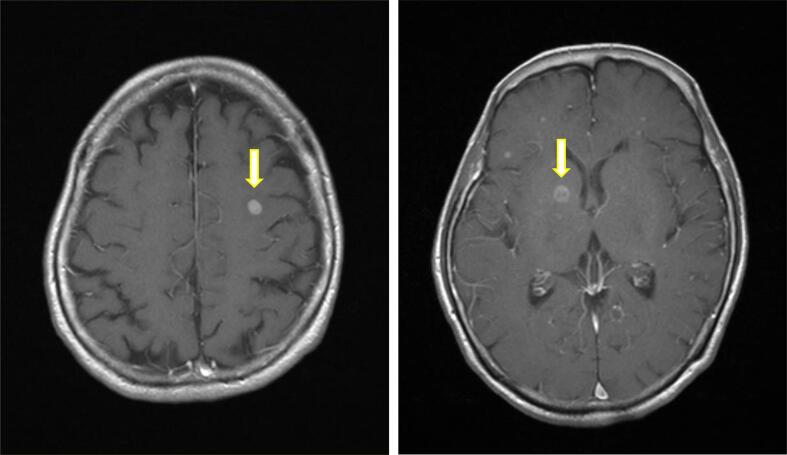
Multiple brain metastases in the white matter of the bilateral cerebral hemispheres (arrow, left and right), the right basal ganglia, and the left dorsal midbrain in Case 1 (axial post-contrast T1-weighted brain MRI).

A 74-year-old woman presented with stage IVB (FIGO 2014) high-grade serous carcinoma (HGSC) involving multiple brain metastases (Fig. 1), multiple lung metastases, multiple lymph node metastases, and rectal invasion. Genetic testing was positive for the germline *BRCA2* (g*BRCA2*) mutation (c.6952C>T (p.Arg2318*)). The patient underwent γ-knife irradiation and received paclitaxel + carboplatin (TC) therapy for nine cycles. However, two years after this initial treatment, she experienced recurrence (peritoneal dissemination) and received six cycles of TC therapy as second-line chemotherapy, followed by maintenance therapy with the poly(ADP-ribose) polymerase (PARP) inhibitor olaparib for seven months. At 45 months post-diagnosis, the disease recurred with peritoneal dissemination and multiple lymph node metastases. Treatment comprised four cycles of liposomal doxorubicin + carboplatin (PLD-C) therapy followed by a single cycle of gemcitabine. The patient died 57 months after the initial diagnosis due to respiratory dysfunction, with a survival period of 57 months post-brain metastasis. Notably, there was no observable recurrence of brain metastases until her death.

### 3.2 Case 2

**Fig. 2:**
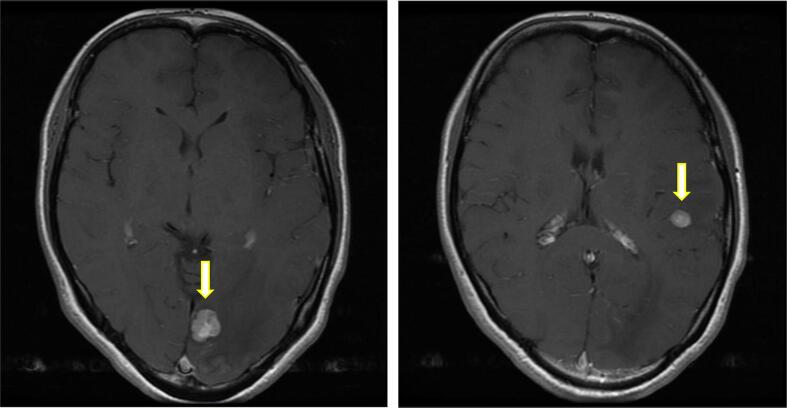
Multiple brain metastases in the left frontal, parietal, and occipital lobes (arrow, left) and the left cerebral hemisphere (arrow, right) in Case 2 (axial post-contrast T1-weighted brain MRI).

A 39-year-old woman presented with stage IIIC (FIGO 2014) HGSC ovarian cancer, exhibiting a negative g*BRCA1/2* mutation. She underwent primary debulking surgery, achieving complete tumor resection. Subsequently, she received six cycles of TC therapy as adjuvant chemotherapy. After 24 months post-diagnosis, recurrence was detected, including multiple brain metastases and lymph node metastases (Fig. 2). Following γ-knife irradiation, she underwent six additional cycles of TC therapy, followed by maintenance treatment with olaparib. As of August 2023, 63 months from initial diagnosis (57 months into olaparib treatment), the patient remained free of recurrence. The survival period post-brain metastasis was 63 months.

### 3.3 Nine case summaries of brain metastasis

During the observation period, our institution treated 598 cases of ovarian cancer (stage III or IV; 314 cases), of which nine cases (1.51 %) presented with brain metastases. The median age at initial diagnosis was 54 years (range: 39–73 years), and the median overall survival among the nine patients with brain metastases from ovarian cancer was 52 months (range: 19–81 months). One case was stage IC, and eight were stage III or IV according to FIGO 2014 criteria. Histologic types were HGSC in eight patients and clear cell carcinoma in one patient. Germline genetic testing was performed in four of the nine patients, revealing a g*BRCA2* mutation in three of them. The median age at diagnosis of brain metastases was 56 years (range: 41–77 years), with a median interval of 24 months (range: 15–64 months) from diagnosis to brain metastases. The median survival time after diagnosis of brain metastases was six months (range: 0–58 months) **(**Table 1**)**. At diagnosis, eight patients were symptomatic (experiencing dizziness, nausea, cognitive dysfunction, etc.), whereas one patient was asymptomatic. Six patients had multiple brain metastases, and three patients had solitary brain metastases. Two patients had brain metastases exclusively, without metastasis to other organs. One patient had brain metastasis at initial diagnosis, whereas others developed brain metastases after recurrence. Treatment after brain metastases included radiotherapy in seven patients, chemotherapy in three patients, and olaparib in two patients (radiotherapy alone: 4 cases; radiotherapy + chemotherapy: 1 case; radiotherapy + chemotherapy + olaparib: 2 cases). No patients received PARP inhibitors before brain metastasis. As presented in Case 1 and Case 2 above, long-term survival of 57 and 63 months was observed when radiotherapy, chemotherapy, and PARP inhibitors were all used as treatment following brain metastasis.

**Table 1:** Nine cases of brain metastases from ovarian cancer included in this study.

Patient age at time of brain metastasis (years)	FIGO stage (2014)	Status at time of brain metastasis	PFI at time of brain recurrence (months)	Tissue type	Number of brain metastases	Extracranial lesions	*BRCA1/2* mutation	Treatment strategy for brain metastases	Survival period after brain metastasis (months)
53	IVB	First recurrence	11	HGSC	Multiple	Peritoneum, lymph node	Unknown	No treatment	0
58	IC	Second recurrence	9	CCC	1	Bone, lung, liver, lymph node	Unknown	Radiotherapy (whole brain)	2
61	IIIC	Second recurrence	27	HGSC	1	None	g*BRCA2*	Radiotherapy (whole brain)	2
53	IIIC	First recurrence	12	HGSC	Multiple	Peritoneum, lung	Unknown	Radiotherapy (whole brain)	3
77	III or IV	First recurrence	3	HGSC	Multiple	Peritoneum, lymph node	Unknown	Radiotherapy (whole brain)	6
56	IVB	First recurrence	13	HGSC	Multiple	None	Unknown	No treatment (patient refused treatment)	10
55	IVA	First recurrence	57	HGSC	1	None	g*BRCA2*	Surgery radiotherapy (stereotactic radiotherapy) chemotherapy (PLD-C)	17
74	IVB	Initial diagnosis	Initial diagnosis	HGSC	Multiple	Pleura, rectum, lymph node	g*BRCA2*	Radiotherapy (γ knife), chemotherapy (TC, PLD-C, GEM), olaparib (maintenance)	57
41	IIIC	First recurrence	15	HGSC	Multiple	Lymph node	*BRCA* wt	Radiotherapy (γ knife), chemotherapy (TC), olaparib (maintenance)	＞63

HGSC, high-grade serous carcinoma; wt, wild type; CCC, clear cell carcinoma; PFI, platinum free interval; PLD-C, doxorubicin + carboplatin; TC, paclitaxel + carboplatin; GEM, gemcitabine.

## 4 Discussion

### 4.1 Risk factors for brain metastasis of ovarian cancer: Germline *BRCA1/2* mutation

Previously, literature reported that 79 % of ovarian cancer brain metastases were HGSC, followed by endometrioid, mucinous, and clear cell histology, with 86 % classified as stage III or IV advanced cancer (Borella et al., 2020). In our present analysis, 88.9 % of patients had HGSC, and 88.9 % were classified as either stage III or stage IV, consistent with previous reports. The *BRCA1/2* gene encodes a protein involved in homologous recombination DNA double-strand break repair. Among nine patients with brain metastases from ovarian cancer at our hospital, four underwent genetic testing, and 75 % tested positive for g*BRCA2* mutation. In Japan, the prevalence of g*BRCA1/2* mutations in advanced ovarian cancer (FIGO stage III or IV) is 24.1 % (*BRCA1*: 16.3 %, *BRCA2*: 7.7 %), and overall, the proportion of g*BRCA1/2* mutations in ovarian cancer (FIGO stage I–IV) is 14.7 % (*BRCA1*: 9.9 %, *BRCA2*: 4.7 %), aligning with prevalence rates in Europe and the United States (Enomoto et al., 2019). A previous study identified g*BRCA1/2* mutation as a risk factor for ovarian cancer brain metastasis, particularly the *BRCA1* mutation, with 68.2 % (15 of 22 patients) showing loss of BRCA1 expression in the tumor (Szarszewska et al., 2019). In our study, 75 % tested positive for g*BRCA1/2* mutation, all of which were g*BRCA2* mutations. As mentioned earlier, the mutation rate of g*BRCA1* is higher than that of g*BRCA2* in ovarian cancer, suggesting that g*BRCA2* mutation may be a risk factor for brain metastasis in ovarian cancer.

### 4.2 Treatment strategies for ovarian cancer brain metastases: The usefulness of PARP inhibitors

In a previous study, the median survival after brain metastasis of ovarian cancer was 10.1 months (Borella et al., 2020). The median survival after brain metastases treated with chemotherapy alone is poor, ranging from 2.5 to 7 months, and multimodal treatment using radiation, surgery, and chemotherapy performed best in each report (Pakneshan et al., 2014, Anupol et al., 2002, Chiang et al., 2012, Marchetti et al., 2016). Among these, Pakneshan et al. reported a favorable median survival of 20.5 months after brain metastases with the combination of whole-brain irradiation, surgery, and chemotherapy (Pakneshan et al., 2014).

Temozolomide, an oral alkylating agent capable of crossing the blood–brain barrier (BBB), is therefore used for chemotherapy in patients with glioblastoma (Hagiwara et al., 2023). However, most chemotherapy agents cannot cross the BBB. Regarding ovarian cancer, paclitaxel concentrations in the brain are very low after intravenous injection, but this can be enhanced by inhibiting p-glycoprotein (Fellner et al., 2002). Carboplatin also cannot pass through the BBB. However, radiation therapy can disrupt the BBB, leading to increased permeability (Hart et al., 2022).

In the present analysis, patients treated with radiation therapy, surgery, and chemotherapy had a favorable survival time of 17 months after brain metastasis, and two patients who received maintenance therapy with olaparib in addition to radiation therapy and chemotherapy had long-term survival of 57 and 63 months. These results not only further support the use of multimodal treatment for brain metastases from ovarian cancer but also suggest that PARP inhibitors may aid in treating brain metastases from ovarian cancer.

PARP inhibitors induce cell death by inhibiting PARP, which is involved in the DNA single-strand repair mechanism. Additionally, the repair of platinum-treated DNA involves a homologous recombination repair mechanism, which is often disrupted in platinum-sensitive ovarian cancers. PARP inhibitors are therefore effective in platinum-sensitive ovarian cancers, especially those with homologous recombination deficiency. Sun et al. measured the BBB permeability of the PARP inhibitors olaparib and niraparib in a mouse model, where niraparib showed greater and more sustained transmigration to the brain than olaparib (Sun et al., 2018). Although not PARP inhibitors, molecularly targeted agents are widely used to treat brain metastases in other cancers. Various tyrosine kinase inhibitors, in particular, have shown high response rates (58–85 %) in brain metastases among *EGFR* mutation-positive non-small cell lung cancer patients, in whom brain metastases are common (Iuchi and Hara, 2019). Due to the rarity of the disease, there are currently no large-scale clinical studies demonstrating the efficacy of PARP inhibitors against brain metastases from ovarian cancer. Table 2 shows case reports of patients with brain metastases from ovarian cancer who were treated with PARP inhibitors (Bangham et al., 2016, Gray et al., 2019, Favier et al., 2020, Morales Vázquez et al., 2020, Tao et al., 2020, Kasherman et al., 2021, Sakamoto et al., 2019, Gallego et al., 2021, Cabitza et al., 2023, Zhang et al., 2022). In a retrospective cohort study by Alizzi et al., which included patients from eight gynecologic cancer centers in the United Kingdom, progression-free survival was significantly improved in 15 patients with ovarian cancer brain metastases treated with PARP inhibitors compared to 14 patients treated with olaparib beforehand (Alizzi et al., 2023). In the present analysis, two patients who received maintenance therapy with olaparib in addition to radiation therapy and chemotherapy for brain metastasis of ovarian cancer showed long-term survival of 57 and 63 months, supporting the utility of PARP inhibitors in the treatment of brain metastasis of ovarian cancer.

**Table 2:** Case studies on the treatment of brain metastases of ovarian cancer using PARP inhibitors.

Authors	FIGO stage (2014)	Tissue type	Number of brain metastases	Extracranial lesions	*BRCA1/2* mutation	Treatment strategy for brain metastases	PFS (months)
Bangham et al., 2016	IVB	HGSC	1	None	*BRCA2*	Surgery, radiotherapy, chemotherapy (CBDCA), olaparib (treatment)	12
Gray et al., 2019	IIIC	LGSC	>2	None	*BRCA1*	Radiotherapy, chemotherapy (GEM + CBDCA), niraparib (maintenance)	22
Favier et al., 2020	IIIC	Unknown	Multiple	Peritoneum	*BRCA2*	Radiotherapy, chemotherapy (CDDP), olaparib (maintenance)	14
Morales Vázquez et al., 2020	IVA	HGSC	2	None	*BRCA1*	Surgery, radiotherapy, chemotherapy (ADR, VP-16, GEM), olaparib (treatment)	9
Tao et al., 2020	IIIC	HGSC	Multiple	Peritoneum	*BRCA2*	Radiotherapy, chemotherapy (CPT-11 + CDDP, CPT-11 + NDP), niraparib (maintenance)	>15
Kasherman et al., 2021	Unknown	HGSC	Multiple	None	*BRCA1*	Radiotherapy, chemotherapy (GEM + CBDCA, GEM + CDDP), olaparib (maintenance)	11
Sakamoto et al., 2019	IIIC	HGSC	Multiple	None	*BRCA1*	Radiotherapy, chemotherapy (TC), olaparib (maintenance)	>18
Gallego et al., 2021	IIIC	HGSC	Multiple	Lymph node	*BRCA1*	Radiotherapy, chemotherapy (GEM + CBDCA), olaparib (maintenance)	>49
Cabitza et al., 2023	IV	HGSC	1	None	*BRCA* wt	Surgery, radiotherapy, chemotherapy (TC), olaparib (maintenance)	>10
Zhang et al., 2022	IIIC	HGSC	1	None	*BRCA* wt	Surgery, niraparib (maintenance)	>29

HGSC, high-grade serous carcinoma; LGSC, low-grade serous carcinoma; wt, wild type; PFS, progression-free survival; CBDCA, carboplatin; CDDP, cisplatin; ADR, adriamycin; VP-16, etoposide; CPT-11, irinotecan; NDP, nedaplatin

Given that only four patients underwent genetic testing, the significance of three out of the four cases being positive for g*BRCA2* mutations while none showed *BRCA1* mutations remains debatable. In this analysis, patients who did not receive chemotherapy after brain metastasis fell into two categories: those unable to undergo aggressive treatment due to old age or low performance status at detection, and those with platinum-resistant tumors where treatment options were limited or not desired by the patient or their family. Although PARP inhibitors can effectively treat brain metastases of ovarian cancer, their use in clinical practice may be limited in some cases.

In conclusion, among patients with brain metastases from ovarian cancer, two out of nine survived longer than 4 years, indicating a high risk of developing brain metastases among patients with g*BRCA2* mutation-positive HGSC. Our results suggest that a multidisciplinary approach including PARP inhibitors may improve the prognosis of ovarian cancer patients with brain metastases.

## Consent

Written informed consent was obtained from the patients for the publication of these case reports and accompanying images (IRB number 20070081). A copy of the written informed consent is available for review by the Editor-in-Chief of this journal upon request.

## CRediT authorship contribution statement

**Yukari Tsuchino:** Writing – original draft, Investigation, Formal analysis, Data curation. **Tatsuyuki Chiyoda:** Writing – review & editing, Writing – original draft, Supervision, Project administration, Methodology, Funding acquisition, Formal analysis, Data curation, Conceptualization. **Mitsuyo Jisaka:** Investigation. **Tomomi Sakamaki:** Investigation. **Momo Hirata:** Investigation. **Mio Takahashi:** Investigation. **Takuma Yoshimura:** Investigation. **Kensuke Sakai:** Investigation. **Michiko Wada:** Investigation. **Wataru Yamagami:** Writing – review & editing.

## Declaration of competing interest

The authors declare that they have no known competing financial interests or personal relationships that could have appeared to influence the work reported in this paper.
